# A resource for functional profiling of noncoding RNA in the yeast *Saccharomyces cerevisiae*

**DOI:** 10.1261/rna.061564.117

**Published:** 2017-08

**Authors:** Steven Parker, Marcin G. Fraczek, Jian Wu, Sara Shamsah, Alkisti Manousaki, Kobchai Dungrattanalert, Rogerio Alves de Almeida, Diego Estrada-Rivadeneyra, Walid Omara, Daniela Delneri, Raymond T. O'Keefe

**Affiliations:** 1 Division of Evolution & Genomic Sciences,; 2 Division of Molecular and Cellular Function, Faculty of Biology, Medicine and Health, The University of Manchester, Manchester M13 9PT, United Kingdom; 3 Department of Microbiology and Immunology, Faculty of Pharmacy, Minia University, Minya 11432, Egypt

**Keywords:** noncoding RNA, snRNA, tRNA, snoRNA, yeast

## Abstract

Eukaryotic genomes are extensively transcribed, generating many different RNAs with no known function. We have constructed 1502 molecular barcoded ncRNA gene deletion strains encompassing 443 ncRNAs in the yeast *Saccharomyces cerevisiae* as tools for ncRNA functional analysis. This resource includes deletions of small nuclear RNAs (snRNAs), transfer RNAs (tRNAs), small nucleolar RNAs (snoRNAs), and other annotated ncRNAs as well as the more recently identified stable unannotated transcripts (SUTs) and cryptic unstable transcripts (CUTs) whose functions are largely unknown. Specifically, deletions have been constructed for ncRNAs found in the intergenic regions, not overlapping genes or their promoters (i.e., at least 200 bp minimum distance from the closest gene start codon). The deletion strains carry molecular barcodes designed to be complementary with the protein gene deletion collection enabling parallel analysis experiments. These strains will be useful for the numerous genomic and molecular techniques that utilize deletion strains, including genome-wide phenotypic screens under different growth conditions, pooled chemogenomic screens with drugs or chemicals, synthetic genetic array analysis to uncover novel genetic interactions, and synthetic dosage lethality screens to analyze gene dosage. Overall, we created a valuable resource for the RNA community and for future ncRNA research.

## INTRODUCTION

Analysis of eukaryotic genomes has revealed that there is widespread, pervasive, transcription that produces a wide range of RNAs (Velculescu et al. 1997; Okazaki et al. 2002; Kapranov et al. 2007; Xu et al. 2009). In addition to the classical ncRNA families like the transfer RNAs (tRNAs), small nuclear RNAs (snRNAs) and small nucleolar RNAs (snoRNAs), new families of ncRNA have been identified. In humans microRNAs (miRNAs), small interfering RNAs (siRNAs), and Piwi-interacting RNAs (piRNAs) are now well known as regulators of gene expression (Ipsaro and Joshua-Tor 2015). In yeast, structured intronic noncoding RNAs (ncRNAs) have been found to be responsible for phenotype maintenance (Hooks et al. 2016). A number of ncRNA mutations have also been associated with disease states (de Almeida et al. 2016; Jenkinson et al. 2016). However, it is the large group of ncRNAs, recently identified from humans to yeast, that are also emerging as key regulators of gene expression (Wu et al. 2012; Quinn and Chang 2016). A relatively small number of these ncRNAs have been ascribed a function, so with their numbers in the thousands many different tools will be required to determine the function of these ncRNAs.

Deletion strain collections in the yeast *Saccharomyces cerevisiae* (*S. cerevisiae*) have proven to be an essential resource for furthering our knowledge of protein encoding gene function and for understanding phenotypic plasticity (Winzeler et al. 1999; Giaever et al. 2002; Delneri et al. 2008; Bivi et al. 2009; Paget et al. 2014). Barcoded deletion strains have allowed numerous informative genome-wide phenotypic screens, synthetic genetic array (SGA) analysis (Costanzo et al. 2010), and chemogenomic screens (Giaever and Nislow 2014). Unfortunately, all classes of ncRNAs are missing from the current barcoded *S. cerevisiae* deletion strain collections.

Besides the known annotated ncRNAs in *S. cerevisiae,* tiling arrays and strand-specific RNA-seq have identified other classes of ncRNA. Comparative analysis of RNA expressed in wild-type yeast strains and in mutant strains where the exosome complex exoribonuclease Rrp6 has been deleted, identified two new classes of ncRNAs, the stable unannotated transcripts (SUTs), and the cryptic unstable transcripts (CUTs) (Xu et al. 2009). Subsequent studies in strains where the cytoplasmic exonuclease Xrn1 has been disrupted, the histone methyltransferase Set2 has been deleted or depletion of the RNA-binding factor Nrd1 identified further classes of ncRNAs, termed Xrn1-sensitive unstable transcripts (XUTs) (van Dijk et al. 2011; Wery et al. 2016), Set2-repressed antisense transcripts (SRATs) (Venkatesh et al. 2016) and Nrd1-unterminated transcripts (NUTs) (Schulz et al. 2013), respectively. Almost half of the identified XUTs, SRATs, and NUTs overlap with a SUT or CUT. While some functions have been ascribed to these novel classes of ncRNAs, a large-scale analysis of their function has been lacking.

Here we describe a collection of 1502 *S. cerevisiae* deletion strains for the functional analysis of ncRNAs. This deletion strain resource includes diploid heterozygote and homozygote strains as well as *MATa* and *MAT*α haploid strains with deletions of annotated snRNAs, snoRNAs, tRNAs as well as the novel SUTs and CUTs (Xu et al. 2009). This deletion strain resource is now available for any interested researcher.

## RESULTS AND DISCUSSION

**FIGURE 1.**
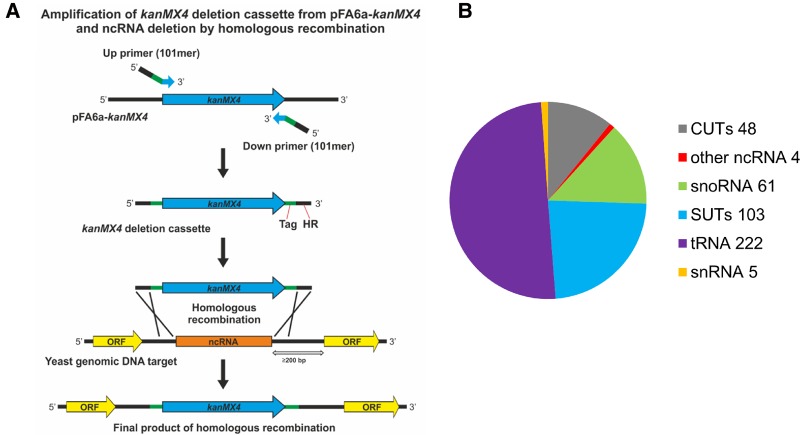
The ncRNA deletion strategy and composition of the ncRNA deletion collection. (*A*) A schematic of the PCR-based ncRNA deletion strategy. The *kanMX4* ncRNA deletion cassette was amplified from the pFA6a-*kanMX4* plasmid using primers “Up primer (101mer)” and “Down primer (101mer).” The primers contain two barcodes (Tag), unique for each ncRNA and genome target homologous region (HR) sequences (upstream of and downstream from the ncRNA) to allow ncRNA disruption by homologous recombination. The *kanMX4* ncRNA deletion cassettes were integrated into the genome at least 200 bp away from the closest open reading frame (ORF) start codon. (*B*) Composition of the ncRNA deletion collection. The “other ncRNA” are *NME1*, *RPR1*, *RUF21*, and *TLC1*.

To allow large-scale analysis of ncRNA function, we have constructed a collection of barcoded ncRNA deletion strains in *S. cerevisiae* covering the majority of the genome. For consistency we used the same methodology used to create the protein deletion collection whereby a gene is replaced with the *KanMX* selectable marker while introducing two unique molecular barcodes that allow parallel analysis of the deletion strains (Giaever et al. 2002). In this case barcodes have been chosen to be complementary with the protein deletion collection and permit the protein and ncRNA deletion strains to be pooled and analyzed together for competitive fitness (Supplemental File 1). We have deleted and barcoded most of the known annotated ncRNAs including tRNAs, snRNAs, snoRNAs, other ncRNAs and the newly identified SUTs and CUTs (Xu et al. 2009) which reside in intergenic regions and do not overlap with neighboring protein coding genes or their promoters (Fig. 1). Specifically, we deleted ncRNAs that were at least 200 base pairs (bp) away from the start codon of a protein coding gene (Supplemental File 1). To construct the deletion strain collection, we first deleted one copy of a ncRNA in a diploid background, thus constructing 428 heterozygous deletion strains in the reference strain BY4743 (Supplemental File 2; Brachmann et al. 1998). Secondly, sporulation and tetrad dissection of the heterozygous strains, as well as direct transformation of haploid BY4741 and BY4742, produced 373 haploid BY4741 (*MATa*) and 370 haploid BY4742 (*MAT*α) strains (Supplemental File 2). A list of the haploid strains which could not have been recovered either after meiosis due to biological issues or due to technical issues are provided in Supplemental Table 1. Finally, by crossing haploid deletion strains of opposite mating types we obtained 331 homozygous ncRNA deletion strains (Supplemental File 2). In total 1502 strains are now available for functional analysis of ncRNAs.

Meiotic studies on the heterozygote ncRNA deletion strains were used to reveal whether a ncRNA was essential in the haploid background. All the already known essential ncRNAs (i.e., snRNAs, snoRNAs, tRNAs) were reconfirmed here (Supplemental File 2). None of the 180 SUTs and CUTs that we deleted were found to be essential in rich medium (YPD) revealing that many of these ncRNAs may function at specific growth stages or under specific environmental conditions.

In the past decade it has become increasingly clear that ncRNAs play key roles in cellular function, in the regulation of other genes and some ncRNAs may also be translated producing functional peptides (Wu et al. 2012; Andrews and Rothnagel 2014; Duncan and Mata 2014; Fu 2014; Smith et al. 2014). Constructing a collection of barcoded ncRNA deletion strains in yeast will now allow researchers to decipher the function of this novel class of genes. Of particular interest are the numerous SUTs and CUTs found in *S. cerevisiae* whose functions are largely unknown (Xu et al. 2009). Recent analysis of the influence of anti-sense SUT transcription on overlapping yeast genes revealed that there is no direct correlation between the presence of an antisense SUT and protein abundance from the overlapping open reading frame (Huber et al. 2016). Here, we have constructed a distinct but complementary resource which includes the deletion of SUTs and CUTs that do not overlap protein coding genes. With this new ncRNA deletion collection, it is possible to study both sense and anti-sense effects of SUTs and CUTs. The function of ncRNAs not overlapping protein coding genes could be through either the ncRNA product itself in *trans* or via the process of ncRNA transcription influencing nearby genes. Examples where transcription of an ncRNA induces or represses the expression of a nearby gene have been found (Uhler et al. 2007; Bumgarner et al. 2009). The known role of ncRNA expression in setting up chromatin modifications that can repress or induce transcription of nearby genes is one mechanism by which ncRNA expression influences nearby genes (Geisler and Coller 2013). The ncRNA deletion strain resource described here, therefore, can be a tool for a more comprehensive expression analysis of genes neighboring ncRNA deletions to ascertain the mechanisms and extent of gene regulation by nearby ncRNA transcription.

The development of barcoded ncRNA deletion strains will also allow whole-genome analysis and competition studies when combined with the barcoded protein deletion collection. Although a tRNA deletion resource has already been developed and has revealed interesting and distinct cellular responses to the deletion of tRNA families (Bloom-Ackermann et al. 2014), it cannot be used for large-scale studies with pooled strains, since the deletion strains are not barcoded. In this respect our collection is a step forward, and will allow comprehensive genome profiling of cellular fitness in different environments (Delneri et al. 2008). The list of common and unique tRNAs deleted in the two collections is provided in Supplemental Table 2. Determining how ncRNAs fit into the genetic networks identified using the protein deletion collection and the synthetic genetic array technology (Baryshnikova et al. 2010) will provide a comprehensive map of protein/ncRNA interactions and elucidate further the global role of ncRNA in the cell. Indeed, scientists have already begun to explore the interplay between ncRNAs and proteins (Kyriakou et al. 2016). Finally, this resource will open up a new facet for large-scale biotechnological applications.

## MATERIALS AND METHODS

### Strains

All strains used are listed in Table 1.

**TABLE 1.**
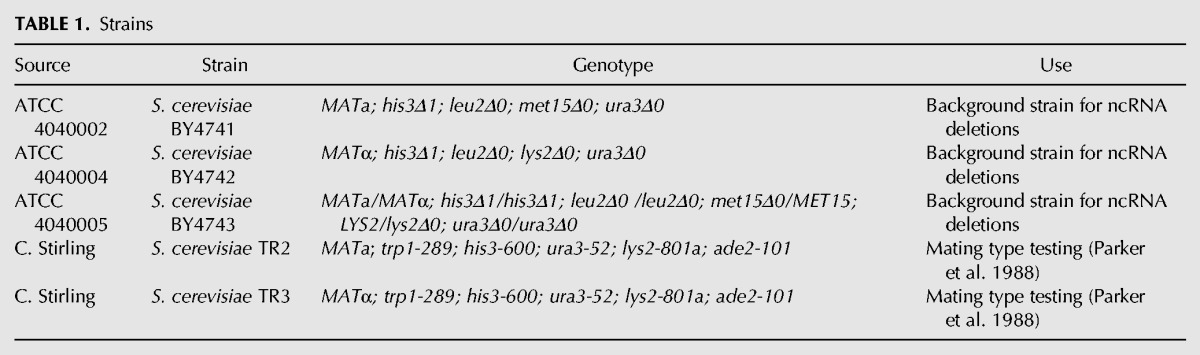
Strains

### Deletion primer design and construction of ncRNA deletion cassettes

A pair of deletion primers for each of the ncRNA genes was designed using the Primer3 software v.0.4.0 (http://bioinfo.ut.ee/primer3-0.4.0/). Primer design was based on a modified version of the *S. cerevisiae* Deletion Project methodology (Winzeler et al. 1999) and primers were purchased from Sigma-Aldrich. All ncRNA deletion cassettes containing *kanMX4* as a marker gene were created in a single PCR step. Each 101 bp forward primer contained a 45 bp sequence homologous to the upstream flank of the ncRNA gene, an 18 bp universal sequence U1 (GATGTCCACGAGGTCTCT), a 20 bp UPTAG barcode and an 18 bp universal sequence (CGTACGCTGCAGGTCGAC) homologous to the 5′ flank of *kanMX4* in pFA6a-*kanMX4*. Reverse primers contained a 45 bp sequence homologous to the downstream flank of the ncRNA gene, an 18 bp universal sequence U2 (CGGTGTCGGTCTCGTAG), a 20 bp DOWNTAG barcode and a 19 bp universal sequence (ATCGATGAATTCGAGCTCG) homologous to the 3′ flank of *kanMX4* in pFA6a-*kanMX4*. UPTAG and DOWNTAG barcode sequences were obtained based on barcodes that would be compatible with the Affymetrix Tag3 array but that had not been used for the YKOv1 or YKOv2 protein deletion collections (http://www-sequence.stanford.edu/group/yeast_deletion_project/deletions3.html). The sequences of the primers and barcodes can be found in Supplemental File 1.

Each deletion cassette PCR was composed of 0.5 µM forward and reverse primers, 50 ng of pFA6a-*kanMX4* and 25 µL of MyTaq Red Mix (Bioline), in a total volume of 50 µL. Cycle conditions were: 1 cycle of 95°C for 2 min, 35 cycles of 95°C for 30 sec, 55°C for 30 sec, and 72°C for 1 min, followed by 72°C for 5 min. Two microliters of PCR were analyzed by electrophoresis to confirm the correct size (∼1.6 kb), and 25 µL was used directly for yeast transformation.

### Generation of ncRNA deletion collection

The ncRNA deletion collection was created using four different approaches. Heterozygous deletions were generated by transformation of the *kanMX4* cassettes into diploid BY4743 to target specific ncRNA genes. Haploid deletions (both *MATa* and *MAT*α) were created by either sporulation of the heterozygote collection or by transformation of BY4741 and BY4742. Homozygote mutants were generated by crossing the opposite mating types of haploid deletion strains.

#### Heterozygote diploid strain creation

Transformation of the deletion cassettes into BY4743 was performed in a 96-well plate, using the methods described by Chu and Davis (2008). Transformants were selected on YPD agar (1% yeast extract, 2% peptone, 2% dextrose, 2% agar) supplemented with 200 mg/L G-418 Disulphate (Melford). After 4 d of incubation at 30°C up to six colonies from each transformation were streaked on YPD-G418 and deletions were confirmed by PCR. PCR confirmation primers can be found in Supplemental File 1.

#### MATa and MATα haploid deletion strain creation

##### Sporulation of heterozygous deletion strains—liquid sporulation method

BY4743 deletion strains were sporulated using a liquid sporulation method (http://www-sequence.stanford.edu/group/yeast_deletion_project/spo_riles). Confirmed heterozygotes were streaked out onto freshly prepared GNA presporulation plates (5% D-glucose, 3% Difco nutrient broth, 1% Difco yeast extract, 2% Bacto agar) and grown for 1 d at 30°C before being streaked out and grown for a second time on a GNA plate. Cells were collected and transferred to 1 mL of supplemented liquid sporulation medium (1% potassium acetate, 0.005% zinc acetate, 0.002% uracil, 0.002% histidine, and 0.003% leucine). The cells were incubated for 5 d at 25°C followed by 3 d incubation at 30°C. Sporulated cells were then dissected. At least 20 tetrads were dissected from each heterozygous strain. Tetrad dissection was performed on YPD agar plates using a Singer instruments MSM 400 or SporePlay microdissector. After incubation for 3 d at 30°C, tetrad dissection plates were replica plated onto YPD agar supplemented with 300 mg/L G418. A 2:2 pattern on YPD plates and no growth on G418 indicated essentiality of the deleted ncRNA gene. For viable knockouts, the two haploids from each tetrad resistant to G418 disulphate were stored. At least 48 haploids for each ncRNA deletion strain were tested for mating type and genotype. Appropriate haploids were then confirmed by PCR.

##### Transformation to obtain haploid deletion strains

For the haploid mutants that could not be obtained by the liquid sporulation method, transformation was performed. The same transformation methodology was used as for the heterozygous diploid strains except that deletion cassettes were transformed into BY4741 and BY4742.

#### Homozygote diploid strain creation

Homozygote diploid deletion strains were created by crossing the *MATa* and *MAT*α strain for each individual ncRNA deletion. The crossing was performed using a Rotor HDA (Singer Instruments) in 96-well microtitre plates with 100 µL of YPD. The cultures were incubated overnight at 30°C and stamped onto SD agar media lacking lysine and methionine (Formedium) and grown for 2 d at 30°C. Strains were then stamped for a second time on the same media and incubated as above. Potential homozygous diploid deletion strains were then transferred into 96-well microtitre plates containing YPD, incubated overnight at 30°C, confirmed by PCR (below) and stored at −80°C.

### Mating type and genotype testing of haploids

#### Mating type testing

Haploids were crossed with both *MATa* (TR2) and *MAT*α (TR3) tester strains (Parker et al. 1988) in 96-well microtitre plates filled with 100 µL YPD and incubated at 30°C overnight. Using a ROTOR HDA, cultures were stamped out on to SD agar media lacking leucine and tryptophan (0.67% Bacto yeast nitrogen base without amino acids, 2% glucose, 2% agar, and 0.155% Yeast synthetic drop-out medium supplement without leucine and tryptophan). Growth resulting from either of the crosses confirms that the haploid was the opposite mating type.

#### LYS2 and MET15 genotyping

After establishing the mating type of haploids, genotyping for lysine and methionine auxotrophic markers was performed.

Using the Rotor HDA, haploid cultures were stamped out onto SD media lacking lysine or SD media lacking methionine. Where possible, haploids with genotypes matching BY4741 and BY4742 were picked. However, if this was not achievable the auxotrophic markers (*LYS2* and *MET15*) of *MATa* and *MAT*α strains were reversed.

### PCR confirmation of strains

All ncRNA deletion strains were confirmed by PCR for the correct gene deletion. Single colonies streaked on YPD-G418 plates were resuspended in 50–100 µL of sterile water and incubated at 95°C for 15 min. Cells were centrifuged at 3000*g* for 5 min and the supernatant was used as a template for the confirmation PCR.

Four sets of primers were used to analyze mutants for deletion of ncRNA genes in accordance with the protocol of Winzeler et al. (1999). Primer pairs ConfA-kanB and ConfD-kanC only generated a PCR product if a correct deletion cassette insertion event occurred. Primers ConfA-ConfB and ConfC-ConfD generated PCR products if a copy (or two copies) of the wild-type allele was intact.

The confirmation PCRs were performed in a total volume of 25 µL and contained 0.5 µM of each primer, 5 µL of DNA template and 12.5 µL MyTaq Red Mix (Bioline). The cycling conditions were as follows: initial denaturation at 95°C for 10 min followed by 35 cycles of 95°C for 30 sec; 57°C for 30 sec; 72°C for 90 sec, and final elongation at 72°C for 5 min. PCR products were analyzed on 1.5% agarose gels.

All confirmed ncRNA mutants were stored in YPD containing 15% Glycerol in both cryovials and 96-well microtitre plates.

## SUPPLEMENTAL MATERIAL

Supplemental material is available for this article.

## Supplementary Material

Supplemental Material
