# Supplementary material for: A resource for functional profiling of noncoding RNA in the yeast Saccharomyces cerevisiae
Source: RNA. 2017 Aug;23(8):1166–71. doi: 10.1261/rna.061564.117 (PMC5513061; doi:10.1261/rna.061564.117)
Supplement: Supplemental Material [file supp_23_8_1166__index.html]

A resource for functional profiling of noncoding RNA in the yeast Saccharomyces cerevisiae — A resource for functional profiling of noncoding RNA in the yeast Saccharomyces cerevisiae — Supplemental Material 

# A resource for functional profiling of noncoding RNA in the yeast *Saccharomyces cerevisiae*

## Supplemental Material

Supplemental Material

- Supplemental\_File\_1.xlsx
- Supplemental\_File\_2.xlsx
- Supplemental\_Table\_1.xlsx
- Supplemental\_Table\_2.xlsx
